# Sugar Functionalized Synergistic Dendrimers for Biocompatible Delivery of Nucleic Acid Therapeutics

**DOI:** 10.3390/polym10091034

**Published:** 2018-09-18

**Authors:** Shuqin Han, Tsogzolmaa Ganbold, Qingming Bao, Takashi Yoshida, Huricha Baigude

**Affiliations:** 1 School of Chemistry & Chemical Engineering, Inner Mongolia Key Laboratory of Mongolian Medicinal Chemistry, Inner Mongolia University, 235 West College Road, Hohhot 010021, China; chem-hshq@imu.edu.cn (S.H.); tsogi001@mail.imu.edu.cn (T.G.); qingming@mail.imu.edu.cn (Q.B.); 2 Department of Bio and Environmental Chemistry, Kitami Institute of Technology, 165 Koen-cho, Kitami, Hokkaido 090-8507, Japan; yoshida@chem.kitami-it.ac.jp

**Keywords:** sugar functionalization, synergistic dendrimer, siRNA delivery, green fluorescence protein, Plk1

## Abstract

Sugars containing cationic polymers are potential carriers for in vitro and in vivo nucleic acid delivery. Monosaccharides such as glucose and galactose have been chemically conjugated to various materials of synergistic poly-lysine dendrimer systems for efficient and biocompatible delivery of short interfering RNA (siRNA). The synergistic dendrimers, which contain lipid conjugated glucose terminalized lysine dendrimers, have significantly lower adverse impact on cells while maintaining efficient cellular entry. Moreover, the synergistic dendrimers complexed to siRNA induced RNA interference (RNAi) in the cells and profoundly knocked down green fluorescence protein (GFP) as well as the endogenously expressing disease related gene Plk1. The new synergic dendrimers may be promising system for biocompatible and efficient siRNA delivery.

## 1. Introduction

Inhibiting the expression of disease related genes at the messenger RNA (mRNA) level has great potential for clinical applications. By the precise design of single or double stranded short nucleic acids sequences, any existing primary or mature RNA transcripts in the cells can be targeted and subsequently hydrolyzed, which is a dramatically advanced drug design compared to the traditional small molecule based drug screen that can only target up to 5% of the total proteins in the cell [1]. There are two related but distinct approaches to target mRNA: the antisense oligonucleotides (ASOs) based approach and the short interfering RNA (siRNA) based approach. While four ASOs (i.e., fomivirsen [2], mipomersen [3], eteplirsen [4], and nusinersen [5]) have been in clinical applications for treatment of diseases, no siRNA therapeutics have been approved by FDA so far [6], although the efficiency of siRNA is significantly higher than any unmodified or chemically modified ASO. Thanks to the highly efficient RNA-induced silencing complex (RISC) [7], siRNA targets and degrades specific mRNA highly efficiently, while single stranded ASOs bind the complementary sequence site of the target mRNA through Watson–Crick base pairing and inactivate it [8]. While chemically stabilized ASOs can be delivered to the cells without carriers, the double stranded siRNAs usually need carriers for protection from nuclease attack, efficient delivery to the cells, and avoiding immune responses, among other reasons. Failed clinical trials for chemically conjugated naked siRNAs such as cholesterol-conjugated siRNA and siRNA-GalNac [9] suggested that the lack of an efficient biocompatible delivery system is one of the bottlenecks for the development of RNA interference (RNAi) therapeutics.

Therefore, the development of an efficient delivery system is urgently needed to realize the clinical application of RNAi therapeutics. Lipid nanoparticle (LNP) is the most advanced technology for systemic siRNA delivery [10]. As low as 0.03 mg siRNA kg-1 ED50 was achieved by an amino lipids delivery system with ionizable head groups [11], which prompted cellular uptake through macropinocytosis and escape from endocytic recycling [12]. An amphiphilic dendrimer bearing ionizable poly(amidoamine) dendrons and a lipid chain with double function of LNPs and polycations successfully delivered siRNA against Hsp27 in a prostate cancer model [13]. Ionizable amphiphilic dendrimers have been formulated for tunable siRNA delivery system to target liver endothelium or tumor in vivo [14]. These examples demonstrate that dendrimers are potential candidates for siRNA delivery, because polycationic dendrimers are ionizable proton sponges that not only complex with nucleic acid and enter the cells, but also initiate endosomal escape for drug release. Furthermore, dendrimers can be readily modified chemically to provide functional groups, such as cell surface targeting moieties.

Carbohydrate functionalized polymers including dendrimers have been explored for siRNA delivery. For example, the first human trial of siRNA therapeutic used cyclodextrin containing polymeric nanoparticles [15,16]. Chitosan has been extensively explored for the potential application of efficient siRNA delivery [17,18]. We reported in vitro and in vivo siRNA delivery by 6-amino-6-deoxy-curdlan and its derivatives including galactose functionalized hepatocytes targeting curdlan nanoparticles [19,20,21,22,23]. These studies demonstrated that carbohydrate containing polymers have great potential for effective siRNA delivery. Previously, we successfully synthesized dendrimers such as sugar functionalized poly-lysine dendrimer [24,25]. Herein, we explored the potential of our sugar conjugated lysine dendrimers as siRNA delivery agents by focusing on the biocompatibility of synergistic dendrimer complexes for in vitro siRNA delivery.

## 2. Materials and Methods

### 2.1. Materials

The following reagents were used as received: *N*,*N*-dimethyl formamide (DMF), chloroform (CHCl_3_), ethyl acetate (EtOAc), n-hexane, sodium sulfate (Na_2_SO_4_), methanol (MeOH), methylene chloride (CH_2_Cl_2_), sodium bicarbonate (NaHCO_3_), sodium hydroxide (NaOH), *N*,*N*,*N*′,*N*′-tetramethyl-*O*-(benzotriazol-1-yl) uronium hexafluorophosphate (Kanto Chemical Co., Inc., Tokyo, Japan), *N*-ethyl-*N*-(1-methylethyl)propan-2-amine (Wako Co., Tokyo, Japan), stearylamine, silver (I) carbonate, *N*,*N*′-dicyclohexyl carbodiimide (DCC), 1-hydroxybenzotriazole (HOBt) (Kishida Chemical Co., Oshaka, Japan), di-tert-butyl-dicarbonate (TCI Co., Tokyo, Japan), hydrobromic acid (acetic acid solution) (Merck KGaA Co., Tokyo, Japan), α-d-cellobiose octaacetate, α-d-Glucose pentaacetate, ethyl 6-hydroxy hexanoate, dimethyl sulfoxide (DMSO), and deuterated solvents (CDCl3, DMSO-*d*_6_ and D_2_O) (Sigma-Aldrich, St. Louis, MO, USA). *N*,*N*-diisopropyl ethylamine (DIEA) were purchased from Wako Pure Chemical Industry Co., Inc. (Tokyo, Japan). Di-Boc-lysine (2) was synthesized according to the previous literature [24].

### 2.2. Measurements

The identities of the compounds were confirmed by the following methods. Both ^1^H-NMR (600 MHz) and ^13^C-NMR (100 MHz) spectra were recorded using a JEOL JNM-ECA 600 (JEOL Ltd., Tokyo, Japan) (and JNM-ECA 920 (JEOL Ltd., Tokyo, Japan)) spectrometer, respectively. The spectra were measured in CDCl_3_ solvents at room temperature, DMSO-*d*_6_ and D_2_O solvents at 40 °C and 4,4′-dimethyl-4-silapentane-1-sulfonate (DSS) as an internal standard. MALDI TOF mass spectra were measured by a Bruker Ultraflex III instrument (Bremen, Germany) with a 337 nm nitrogen laser. The dendrimer sample solutions (1 μL, 1 mg/100 μL of MeOH:THF = 1:1) and the matrix solution (1 μL, 2,5-dihydroxybenzoic acid of 2 mg/100 μL MeOH:THF = 1:1) were applied to the 24 × 16 well ground steel MALDI probe (Bruker Daltonics K.K., Yokohama, Japan). The dendrimers sample solution (1 μL, 1 mg/100 μL of 0.1% (*v*/*v*) TFA in ACN:H_2_O = 2:1) and the matrix solution(1 μL, 2,5-dihydroxybenzoic acid of 2 mg/100 μL of 0.1% (*v*/*v*)TFA in ACN:H_2_O = 2:1) were applied to the 24 × 16 well ground steel MALDI probe. The sample was dried by air evaporation and then the nitrogen laser was irradiated to each sample to obtain the corresponding mass spectrum.

### 2.3. Synthesis

Preparation of AL-NH_2_ dendrimers: Stearylamine (1.08 g, 4 mmol) and di-boc lysine (2.2 g, 4.2 mmol) was stirred in DMF (anhydrous, 30 mL) under N2 atmosphere. The mixture was cooled in an ice bath, and DIPEA (1.4 mL 8 mmol) and HBTU (1.59 g, 4.2 mmol)/HOBt (0.57 g, 4.2 mmol) was added, followed by stirring for 24 h at room temperature. The solvent (DMF) was removed and the residue was dissolved in ethyl acetate and then washed successively with 15% NaCl solution, 5% aqueous citric acid solution, NaHCO_3_ saturation solution, and water several times. The ethyl acetate layer was then evaporated to give alkyl chain lysine (yield: 94%) after purification by silica gel column chromatography. The alkyl chain conjugated lysine (1.2 g, 2 mmol) was vigorously stirred in a mixture solution of TFA and dry DCM for 30 min. Then the solvent was removed, diethyl ether was added. The precipitate was collected by centrifugation and washed extensively with anhydrous diethyl ether. After drying, the deprotected alkyl chain conjugated lysine (TFA salt) was used immediately for the next condensation without purification. Condensation of the deprotected alkyl chain conjugated lysine and di-boc lysine (2.1 g, 4 mmol) with DIEA (0.7 mL 4 mmol) and HBTU/HOBt (0.8 g, 4.2 mmol/0.29 g, 4 mmol) in DMF gave the first generation alkyl chain lysine dendrimer (ALDG1) a 92% yield after column chromatography purification. The second generation and the third generation alkyl chain conjugated lysine dendrimer AL-NH_2_ were obtained by the same procedures in 86% and 79% yields, respectively.

Synthesis of AL-Glu-dendrimer 2nd generation: The deprotected ALDG2 TFA salt (0.36 g, 0.17 mmol) and 1-*O*-(6-carboxyl hexanoyl) glucoside (0.41 g, 1.4 mmol) was stirred in anhydrous DMF (20 mL under a nitrogen atmosphere. DCC/HOBt (0.32 g, 1.6 mmol/0.2 g, 1.6 mmol) was added, followed by stirring for five days at room temperature. After the mixture was filtered, the DMF was removed under reduced pressure. The mixture was dissolved in water and then dialyzed against deionized water for two days to give AL-glucose-dendrimer 2st generation (AL-Glu) in 73% yield after freeze-drying.

### 2.4. Electrophoretic Mobility Shift Assay

An electrophoretic mobility shift assay was used to assess the incorporation of the plasmid DNA by the AL-NH_2_ dendrimer or synergistic AL-NH_2_/AL-Glu dendrimers into the complexes. The dendrimers/pDNA complex was prepared at various N/P ratios (0.5–1.5) and H_2_O was added up to 10 μL in total volume. After the complexed mixtures were incubated at RT for 20 min, 2 μL of 6× loading buffer was added to the mixture, and then electrophoresed on a 2% (*w*/*v*) agarose gel in 1XTBE buffer for 30–40 min at 100 V. After the gel was stained with 0.5 µg/mL ethidium bromide for 15–20 min, the location of siRNA in the gel was analyzed on a UV illuminator using a Gel Logic 212 PRO imaging system (Carestream, Toronto, ON, Canada).

### 2.5. In Vitro siRNA Delivery

HeLa cells and HepG2 cells (ATCC, Manassas, VA, USA) were maintained at 37 °C with 5% CO_2_ in DMEM supplemented with 10% fetal bovine serum (Hyclone Laoratories, Inc, Logan, UT, USA), 100 U mL^−1^ penicillin and 100 µg mL^−1^ streptomycin. For cytotoxicity assay, cells were plated onto 96-well plates at 5000 cells per well in 100 μL of culture medium. 24 h after plating, 100 μL of the synergistic dendrimer solutions (concentrations: 10, 20, 40, 60, 100 nM) to be tested, was added to the cells (in triplicate) and incubated as described above. After a 24 h exposure period, cell viability was assayed using the MTT test. For in vitro transfection experiment, siRNA duplex targeting GFP was designed as previously described [26]. Plk1 siRNA was custom synthesized by TaKaRa Biotechnology Co., Ltd. (Dalian, China). The sequence of Plk1 siRNA: antisense strand: AGACUCAGGCGGUAUGUGCUU; sense strand: GCACAUACCGCCUGAGUCUUU [27]. Cells (2.5 × 105 cells per well) were seeded into 12-well plates at the day before transfection. Cells were transfected with 0.5 mL per well of siRNA (50 nM) complexed to the synergistic dendrimers (3 μg/mL) for 4.0 h at 37 °C. To determine siRNA levels in cell culture after siRNA treatment, total RNA was extracted with TRIZOL (Invitrogen, Carlsbad, CA, USA). The expression of mRNA was measured using iScriptTM Reverse Transcription Supermix for RT-qPCR and iTaqTM Universal SYBR Green Supermix (Bio-Rad, Hercules, CA, USA) for quantitative PCR. The sequences of primers used for RT-qPCR were as following: human -actin, forward: 5’-ccaaccgcgagaagatga-3’, reverse: 5’-ccagaggcgtacagggatag-3’, human Plk1, forward: cacagtgtcaatgcctcca, reverse: ttgctgacccagaagatgg.

### 2.6. Statistical Analysis

Data were presented as mean ± SD. Statistical analysis was performed by one-way analysis of (ANOVA) followed by Dunnett’s multiple comparisons test using GraphPad Prism 7.0 (GraphPad Software, San Diego, CA, USA). A value of *p* < 0.05 was considered statistically significant.

## 3. Results

**Scheme 1:**
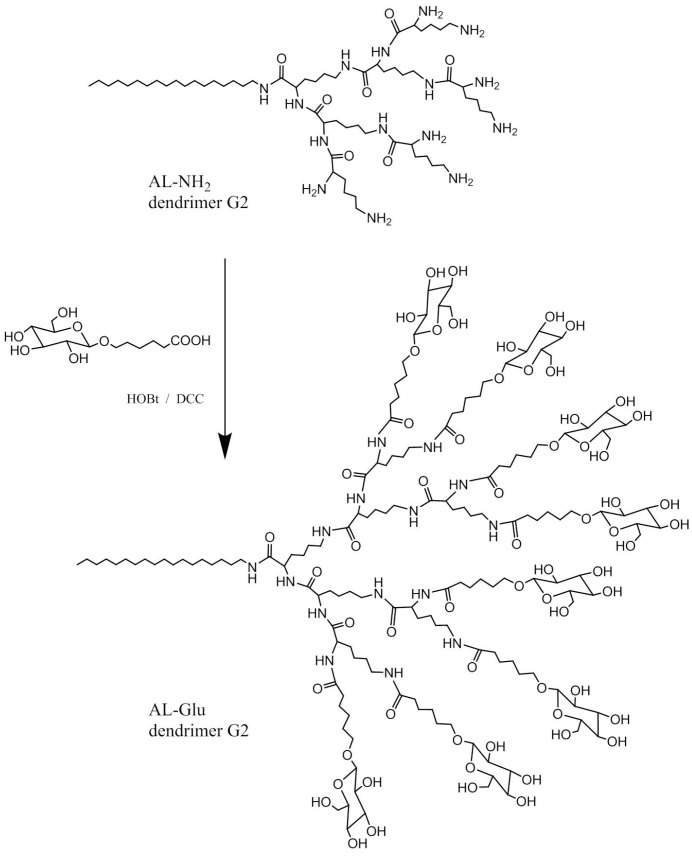
Schematic illustration of the synthesis of fatty acid chain and glucose functionalized lysine dendrimer AL-Glu.

**Figure 1:**
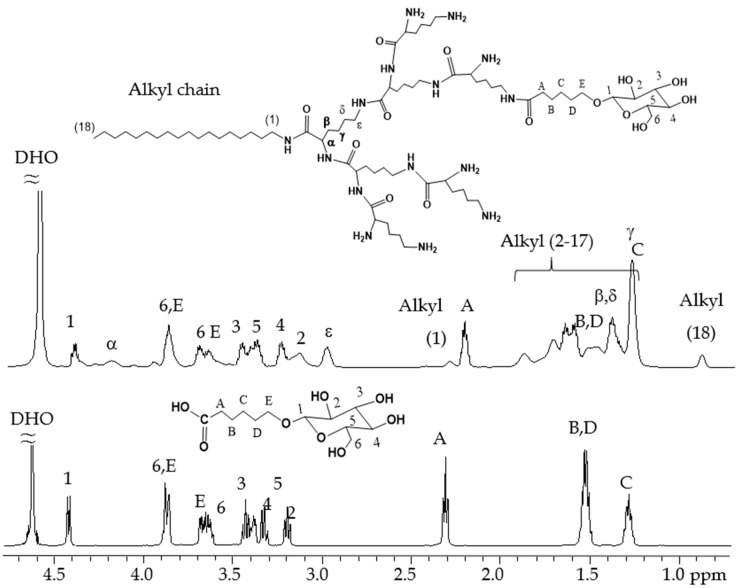
^1^H-NMR spectra of glucose derivative and AL-Glu dendrimer in D_2_O.

**Figure 2:**
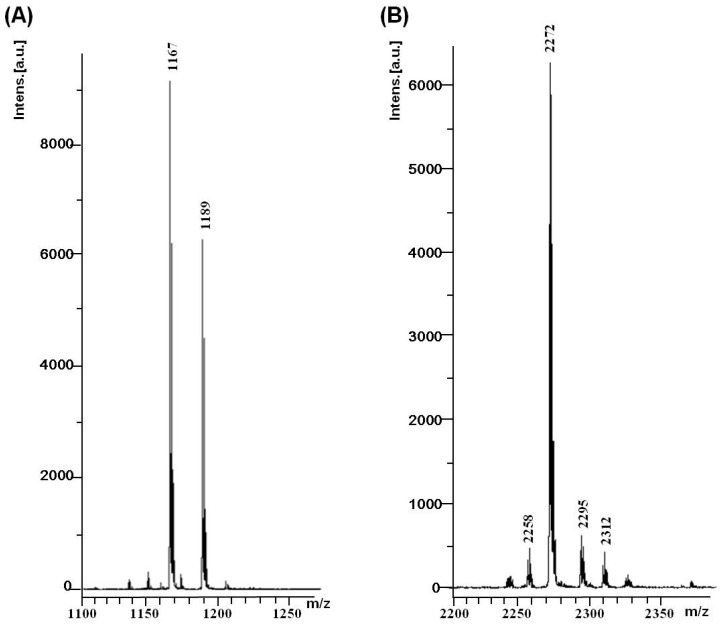
MALDI TOF MS analysis of AL-NH_2_ and AL-Glu. (**A**) AL-NH_2_ dendrimer, calculated mass: 1167; found: 1167 and 1189 (M + Na); (**B**) AL-Glu dendrimer, calculated mass for fully substituted molecule: 3367, found: 2272–2312 (near [M-glucose unit 4] ion).

In order to create a biocompatible siRNA delivery system based on ionizable polyamine, we designed a double functionalized low generation lysine dendrimer as follows: stearylamine was conjugated at the C terminal of the lysine dendrimer generation 2 (G2), and the N terminal amines of the dendrimer were coupled with glucose through a short linker (Scheme 1). The rationale for such a design is based on the fact that the dendrimer, like all other polycations, tends to aggregate with nucleic acids in solution. Incorporation of stearyl chain in the structure may facilitate the formation of amphiphilic lipoplex, which is more stable and efficient for siRNA binding as well as cellular uptake. Meanwhile, the glucose units on the dendrimer may also contribute to the cellular entrance of the polyplex while increasing the biocompatibility and lowing the cytotoxicity. To conjugate glucose to the dendrimer, we first prepared a carboxyl functionalized glucose derivative according to the previously reported method [24]. The product of the next step synthesis, glucose functionalized dendrimer G2 (denoted AL-Glu), was first characterized by ^1^H-NMR analysis (Figure 1). A comparison between glucose derivative (glucose with short linker) and the last product confirmed the coupling of the glucose units to the dendrimer. The stearylamine conjugated dendrimer (denoted AL-NH_2_) was synthesized with appreciable yields, and the perfect match of the mass between the designed molecule and the obtained product confirmed that the synthesis was successful (Figure 2A). However, analysis of AL-Glu dendrimer by MALDI-TOF mass revealed that out of 8 terminal amino groups of AL-NH_2_ dendrimer, 4 reacted with the glucose derivative (Figure 2B). Therefore, the final product bears 4 glucose units and 4 free amines at the N terminal. The free amines are probably alpha amine of lysine residues, because alpha amines may be less reactive, considering the steric effects to the relatively large glucose derivative.

**Figure 3:**
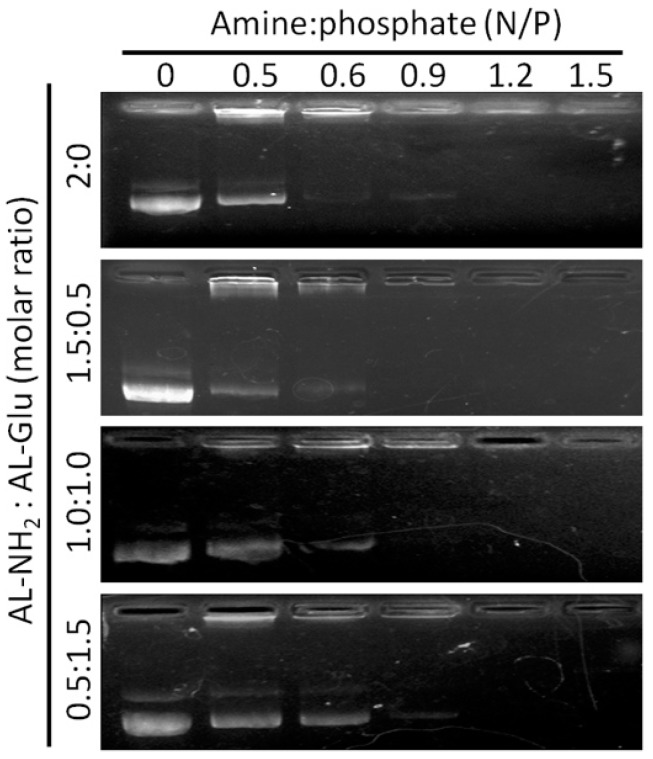
Nucleic acid binding assay of synergistic dendrimers. AL-NH_2_ dendrimer, or synergistic AL-NH_2_/AL-Glu dendrimers at various ratios were incubated with a plasmid DNA for 20 min and applied to gel electrophoresis.

Next, we evaluated the DNA binding ability of AL-NH_2_ alone, as well as the synergistic dendrimers comprised of AL-NH_2_ and AL-Glu at molar ratios of 3:1, 1:1, and 1:3. In DNA gel retardation assay, AL-NH_2_ showed excellent DNA binding ability, with a N/P ratio 0.6 achieving complete retardation of a plasmid DNA. The synergistic dendrimer (3:1), a N/P ratio of 0.9 was required for a total blocking of DNA electrophoresis. For synergistic dendrimer (1:3), a higher N/P ratio of 1.2 was needed for an efficient bind to plasmid DNA. Nevertheless, the formulation of AL-NH_2_ and AL-Glu dendrimers at above tested ratios was not a significant detriment to the nucleic acid binding efficiency of the dendrimers (Figure 3). Therefore, synergizing of lysine dendrimer with and without glucose conjugation may not affect the nucleic acid transfection efficiency of the lysine dendrimer.

**Figure 4:**
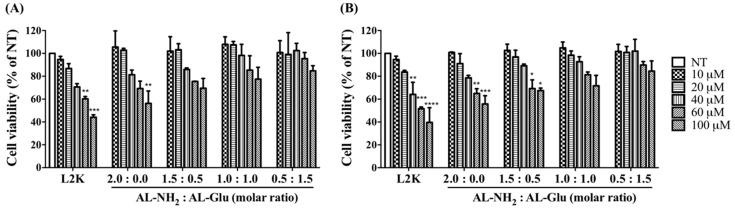
MTT assay for synergistic dendrimers. HeLa cells (**A**) and HepG2 cells (**B**) were treated with increasing concentrations of synergistic dendrimers containing various ratios of AL-NH_2_ and AL-Glu, respectively. After 24 h, cell viability was measured by MTT assay. L2K, lipofectamine 2000. Each value represents the mean ± SD (*n* = 4). The symbol “*” indicates significant different compared to non-treated (NT). *: *p* < 0.05, **: *p* < 0.01, ***: *p* < 0.005 and ****: *p* < 0.001 compared with NT group.

Polycations including high generations of poly-lysine dendrimers induce considerable cellular damage at high concentrations. To evaluate the possible cytotoxicity of the synergistic dendrimers, we treated cells with either AL-NH_2_ alone or synergistic dendrimers with varying ratio of AL-NH_2_ and AL-Glu. Two cell lines (i.e., HeLa cells and HepG2 cells) were treated with increasing concentration of the dendrimers for 24 h. The dendrimers, either alone or combined together, did not induce apparent cell death at lower concentrations. A the highest concentration tested (100 nM) about 56% of HeLa (*p* < 0.005) and 55% HepG2 (*p* < 0.001) cell survival was observed for AL-NH_2_; synergizing with AL-Glu compromised the cytotoxicity of AL-NH_2_ dose dependently: at a 3:1 synergizing ratio, 70% Hela (*p* < 0.05) and 67% HepG2 (*p* < 0.05) cells survived; at a 1:1 synergizing ratio, 77% Hela (*p* > 0.05) and 71% HepG2 (*p* > 0.05) cells survived; at a 1:3 synergizing ratio, about 85% Hela (*p* > 0.05) and 85% HepG2 (*p* > 0.05) cells survived (Figure 4A). However, HepG2 cells appeared more susceptible to the synergistic dendrimers, since lower cell survival rates were observed (Figure 4B). Notably, the concentrations used for the MTT test were significantly higher than the actual concentrations used for nucleic acid transfection (Materials and methods). These data indicated that, synergizing with glucose functionalized dendrimer significantly reduces the cytotoxicity of lysine dendrimer and may increase biocompatibility of the dendrimer.

**Figure 5:**
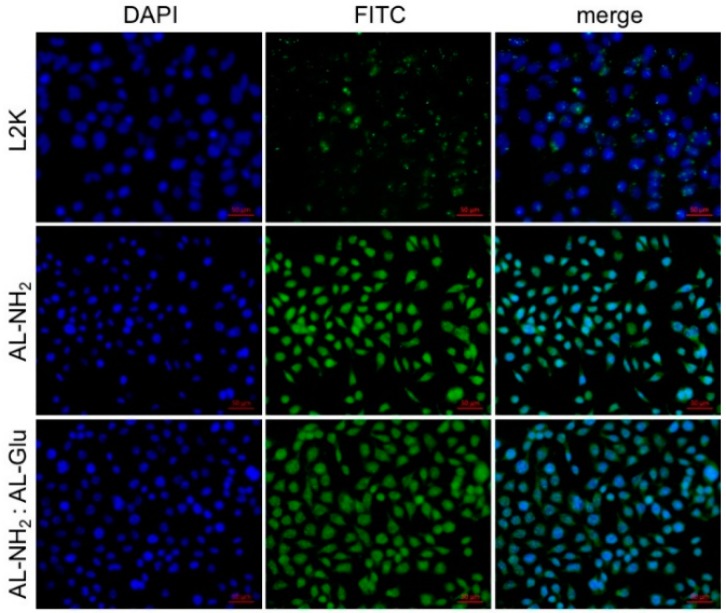
Nucleic acid delivery assay. AL-NH_2_ dendrimer or synergistic AL-NH_2_/AL-Glu dendrimer (1:3) were complexed with a FITC-labeled siRNA, respectively. HepG2 cells were treated with the complexes for 4 h and the fluorescence signal was observed under fluorescence microcopy. The Nucleus was stained with DAPI.

Next, we assessed the efficiency of cellular entrance of the synergistic dendrimers. To do this, we first complexed a FITC-labeled siRNA with either AL-NH_2_, or synergistic dendrimers, and treated HeLa cells with complexes. After 4 h, cell nuclei were stained with DAPI and the cells observed under confocal microscope for fluorescence imaging. A sharp fluorescence signal was observed in the cytosol of cells treated with AL-NH_2_/FITC-siRNA complex. A strong FITC signal was also observed in the cells treated with the synergistic (1:3) AL-NH_2_/AL-Glu/FITC-siRNA, although the signal was slightly weaker compared to the previous ones (Figure 5), demonstrating that the synergistic dendrimer maintains the most transfection efficiency of the original AL-NH_2_ dendrimer.

**Figure 6:**
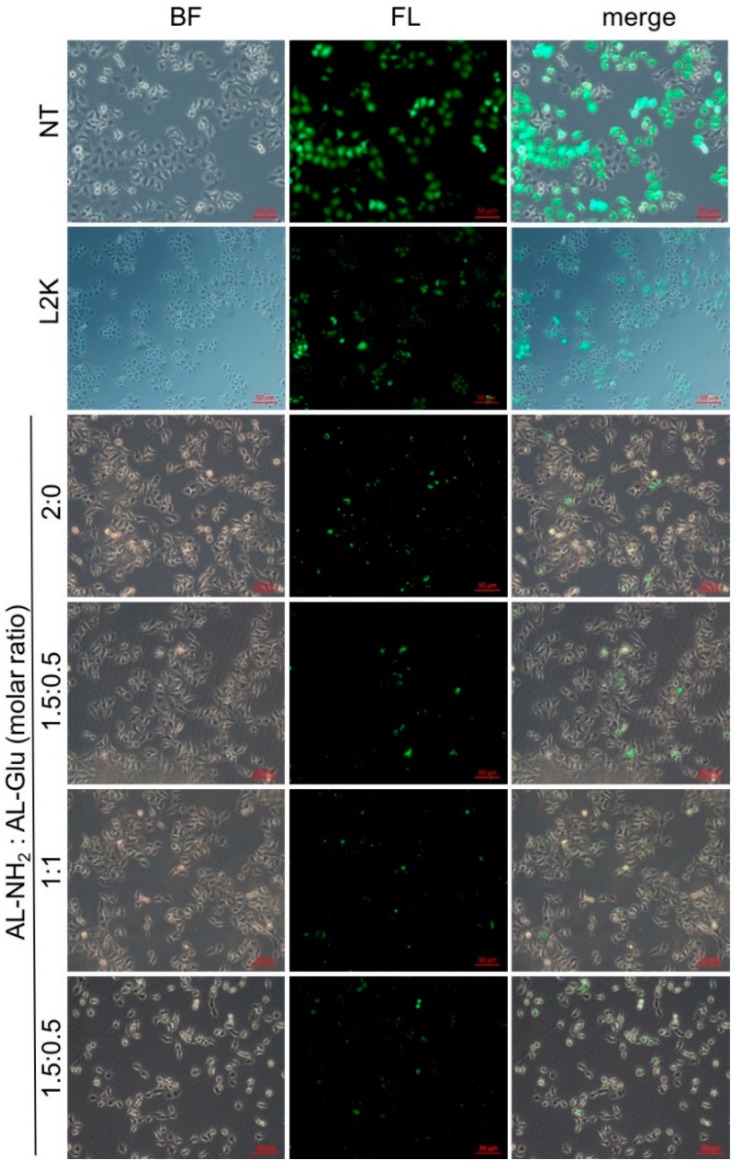
siRNA delivery efficiency of the synergistic dendrimers. AL-NH_2_ dendrimer or synergistic AL-NH_2_/AL-Glu dendrimer swere complexed with siGFP, respectively. HeLa cells stably expressing GFP were treated with the complexes for 24 h and the fluorescence signal was observed under fluorescence microcopy.

**Figure 7:**
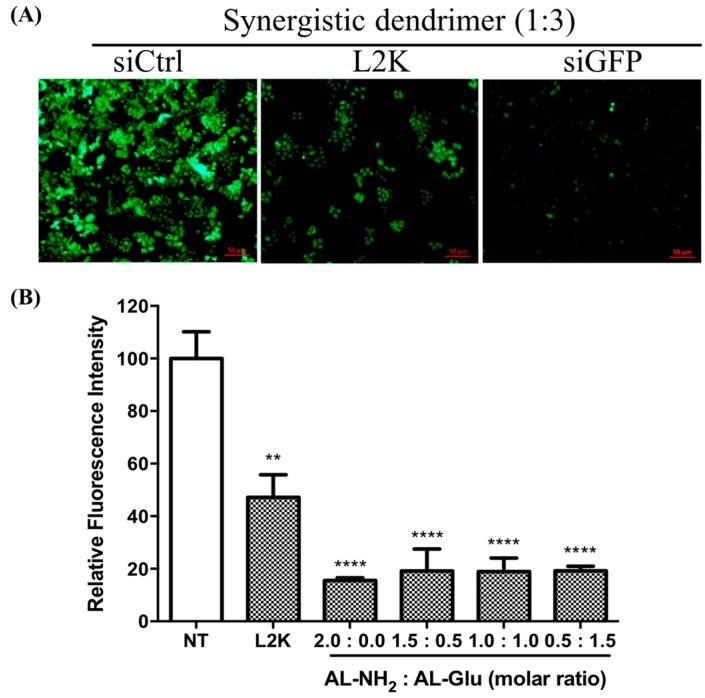
Measurement of GFP protein level of HeLa cells after transfection. HeLa cells stably expressing GFP were treated with siGFP/dendrimer complex for 24 h. (**A**) Confocal images of GFP expressing cells treated with either a non-targeting siRNA (siCtrl), or siRNA against GFP complex to lipofectamine 2000 (L2K) or complex to a synergistic dendrimer (1:3); (**B**) Total protein was extracted and the fluorescence signal was measured. Each value represents the mean ± SD (*n* = 6). The symbol “*” indicates significant different (*p* < 0.05) compared to non-treated (NT). *: *p* < 0.05, **: *p* < 0.01*,* ***: *p* < 0.005 and ****: *p* < 0.001 compared with NT group.

Since glucose modified dendrimer can efficiently deliver dye labeled siRNA to the cells, we next tested whether or not the siRNA escapes from the endosome and initiates RNAi activity in the cytoplasm. To do this, we transfected HeLa cells stably expressing green fluorescence protein (GFP) with siGFP complexed to the dendrimers. After 24 h, the GFP expression level was evaluated by fluorescence microscope observation. Compared to the no-treated cells, GFP expression was significantly reduced by AL-NH_2_ alone, as well as the synergistic dendrimer with all tested mixing ratios (Figure 6). To quantify the GFP knockdown efficiency, we measured the fluorescence intensity of the total proteins extracted from non-treated cells and the siRNA transfected cells. In the cells treated with AL-NH_2_/siRNA, a 85% (*p* < 0.0001) decrease of GFP level was observed (Figure 7). In the cells treated with siRNA complexed to the synergistic dendrimers, about 81% (*p* < 0.0001) GFP was knockdown, indicating that the synergic dendrimers have equal efficiency of siRNA delivery with the original unmodified lysine dendrimer.

**Figure 8:**
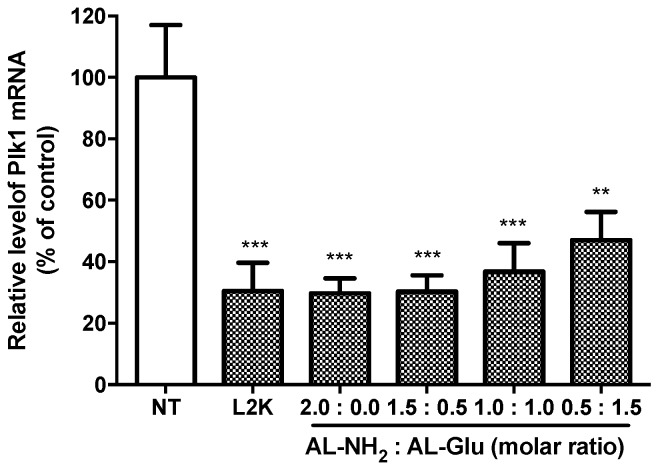
Quantification of siRNA delivery efficiency of the synergistic dendrimers. HepG2 cells were treated with siPlk1 complexed to the dendrimers. After 24 h, total RNA was isolated from the cells and the mRNA level of Plk1 was quantified by RT-qPCR. Each value represents the mean ± SD (*n* = 4). The symbol “*” indicates significant different (*p* < 0.05) compared to non-treated (NT). *: *p* < 0.05, **: *p* < 0.01, ***: *p* < 0.005 and ****: *p* < 0.001 compared with NT group.

To quantify the siRNA delivery efficiency of the synergic dendrimers at the mRNA level, we treated HepG2 cells with synergic dendrimer/siRNA complexes. We chose a disease related gene Plk1 as the silencing target. After 24 h, we extracted total RNA from the cells and analyzed the Plk1 mRNA level by RT-qPCR. A 70% (71.66 ± 10.01; *p* < 0.0005) decrease of Plk1 mRNA was achieved in the cells transfected with AL-NH_2_ (Figure 8). Remarkably, the synergic dendrimer 3:1 induced equally efficient RNAi, indicating that synergizing AL-NH_2_ with glucose functionalized poly-lysine not only significantly lower the potential cytotoxicity but also maintain equal siRNA delivery efficiency. However, in synergic dendrimer 1:1 and 1:3, decreased RNAi against Plk1 was observed (64% (64.18 ± 10.76; *p* < 0.001) and 55% (54.4 ± 10.74; *p* < 0.005) knockdown, respectively), suggesting that the increasing ratio of AL-Glu may affect the transfection efficiency of the resulting synergic dendrimers.

The advantage of our glucose functionalized dendrimer compared to liposome and other dendrimers (such as PAMAM) are the robust synthetic routes, remarkable affinity to small interfering RNA, significantly lower cytotoxicity, as well as flexibility for synergizing to modulate the efficiency of cellular uptake.

## 4. Conclusions

The poly-lysine based glucose functionalized dendrimer was prepared to test the biocompatibility and siRNA delivery efficiency in vitro. The complexes of dendrimers with and without sugar conjugation provided synergistic dendrimer systems with reduced cytotoxicity while maintaining the transfection efficiency. Stably expressing GFP was knocked down by the synergistic dendrimers at both the mRNA and protein levels. Moreover, a disease related gene Plk1 was also significantly knocked down in HepG2 cells by the synergistic dendrimers. Collectively, our data demonstrated that the synergistic dendrimers may be a promising biocompatible system for siRNA delivery.
